# Intractable hiccup leading to life-threatening malnutrition: case report

**DOI:** 10.3389/fnut.2025.1729025

**Published:** 2026-01-20

**Authors:** Kinga Miaśkiewicz, Maria Janiak, Marcin Folwarski

**Affiliations:** 1 Department of Paediatrics, Gastroenterology, Allergology and Nutrition, Medical University of Gdańsk, Gdańsk, Poland; 2 Department of Gastroenterology and Hepatology, Faculty of Medicine, Medical University of Gdańsk, Gdańsk, Poland; 3 Department of Clinical Nutrition and Dietetics, Medical University of Gdańsk, Gdańsk, Poland; 4 Home Enteral and Parenteral Nutrition Unit, Nicolaus Copernicus Hospital, Gdańsk, Poland

**Keywords:** enteral nutrition, gastroesophageal reflux disease, intractable hiccups, nutritional support, PEG-PEJ, severe malnutrition

## Abstract

Intractable hiccups, lasting over a month, can severely impair quality of life and, in rare cases, significantly limit the ability to maintain adequate oral nutritional intake. We report a 63-year-old male with a 5-year history of intractable hiccups, 30 kg weight loss (37%), muscle wasting, and functional decline. Extensive diagnostics found no clear cause, and multiple therapies failed. Severe oral intake difficulties prompted initiation of enteral nutrition via PEG with jejunal extension (PEG-PEJ), resulting in a 15 kg weight gain and functional recovery over 6 months. One year later, during a hiccup relapse, the jejunal tube was dislodged. With gastric access intact, we transitioned to gastric feeding, which was well tolerated despite persistent hiccups. This case underscores that malnutrition may be a significant and underrecognized complication in chronic hiccups, with no established nutritional strategies described. Our experience suggests gastric feeding may be feasible in such patients. Further research is needed to guide management.

## Introduction

Hiccups are sudden involuntary contractions of the diaphragm and intercostal muscles followed by abrupt glottic closure, producing the characteristic “hic” sound (1). Numerous factors can contribute to hiccups, including organic causes, psychogenic triggers, idiopathic origins, and those induced by medications (2). Moreover, intractable hiccups may indicate the presence of serious underlying medical issues (3). While most hiccup episodes resolve within 48 h, those lasting more than 1 month are classified as intractable (1). Chronic hiccups can severely impair oral intake, sleep, communication, and overall quality of life (1, 4). Fatigue, dehydration, and weight loss may occur, and in severe cases, can result in death (5, 6). We present a case of a 63-year-old man who was referred to the Home Nutrition Outpatient Clinic due to severe malnutrition that had progressively worsened because of intractable hiccups.

## Case report

A 63-year-old man was referred to our Home Nutrition Outpatient Clinic for management of severe malnutrition following a five-year history of intractable hiccups. He reported progressive postprandial vomiting and inability to maintain adequate oral intake, resulting in a 30 kg (37%) weight loss. On assessment, his body weight was 51 kg with a height of 170 cm, corresponding to a BMI of 17.6 kg/m^2^, classifying him as severely malnourished according to GLIM criteria (7). Physical examination revealed profound muscle wasting, generalized weakness, poorly developed subcutaneous fat stores, and significant difficulty in walking due to reduced muscle strength. Bioimpedance analysis was not performed due to equipment unavailability; however, clinical findings were consistent with severe sarcopenia.

Hiccups had a considerable impact on the patient’s quality of life. Due to the persistent nature of his hiccups, he experienced difficulties in speaking and communication. He also suffered from depression, low mood, and fatigue. Hiccups occurred both during the day and at night. The only relief he found was by triggering his vomiting reflex with deep insertion of a toothbrush into his throat, which alleviated the symptoms for approximately 1 to 1.5 h and allowed him to fall asleep.

His medical history included gastroesophageal reflux disease (GERD), hypertension, hypercholesterolemia, gout, leukoaraiosis, and previously excised nasal squamous cell carcinoma. He had a 40-year smoking history (now e-cigarette use) and a history of alcohol abuse, consuming 1–2 beers daily. An extensive diagnostic workup was conducted 1 year prior to his admission to the Nutrition Unit, during hospitalization in the Gastroenterology and Hepatology Department. Laboratory investigations excluded metabolic causes, including electrolyte disturbances and diabetes. Gastroscopy confirmed gastropathy and reflux esophagitis. Chest CT and abdominal MRI excluded thoracic and abdominal structural lesions. Neurological evaluation, including head CT and MRI, demonstrated an elongated left vertebral artery compressing the ventral-left medulla oblongata, and nerve conduction studies revealed axonal damage to the left phrenic nerve with no primary cause (no compression or injury) and considered as a probable result of chronic hiccup. Psychiatric assessment excluded psychogenic causes. A complete list of diagnostic tests performed and conditions excluded is provided in Table 1. Despite this comprehensive evaluation, no single definitive treatable etiology was identified. Prior treatments included proton pump inhibitors (PPIs, used continuously from symptom onset and ongoing), haloperidol (3 months, discontinued due to lack of sustained effect), baclofen (3 weeks, discontinued), carbamazepine (1 month, discontinued due to lack of response), and metoclopramide (1 month, discontinued as it failed to control vomiting). Non-pharmacological approaches, including the Forced Inspiratory Suction and Swallow Tool (FISST) (27), provided only transient relief. Phrenic nerve ablation was discussed with the patient as a potential intervention. However, after careful consideration of the associated risks, including permanent diaphragmatic paralysis with potentially significant respiratory compromise, the patient declined this procedure.

**Table 1:** Diagnostic evaluation tests done to identify the primary cause of hiccups.

Diagnostic testing	Test results	Excluded causes
Laboratory testing		
Electrolytes, renal function, glucose, hematology	No abnormalities	Hyponatremia, hypokalemia, hypocalcemia, hypocapnia, renal failure, diabetes mellitus, uremia, leukemia
Abdominal evaluation		
Physical examination	No abnormalities	Hepatomegaly, splenomegaly, prostatic disorders
Gastroenterology consultation	No abnormalities	General GI pathology
Gastroscopy	Gastropathy; reflux esophagitis	Achalasia, esophageal obstruction/cancer, stomach distension, ulcers, upper GI bleeding, atony
Colonoscopy	Sigmoid colon polyps, including one with high-grade dysplasia	Bowel obstruction, IBD, lower GI bleeding
Barium swallow examination	Tertiary peristaltic waves hindering bolus passage and causing contrast regurgitation; preserved gastric peristalsis with normal gastric emptying; thickened mucosal folds in the supradiaphragmatic esophagus producing elongated filling defects; markedly thickened gastric mucosal folds; distorted duodenal bulb with an ulcer niche surrounded by edematous mucosa (findings not confirmed on gastroscopy)	Hiatus hernia, esophageal obstruction
MRI of the abdomen	No abnormalities	Eventration, diaphragmatic tumors, cholecystitis, pancreatitis, abdominal abscess, intra-abdominal tumors, hydronephrosis
Chest evaluation		
Physical examination	No abnormalities	Pleural effusions, obvious trauma
ECG	No abnormalities	Myocardial ischemia, pericarditis
Chest X-ray	No abnormalities	Pneumonia, pleuritis, large effusions, carcinoma, TB, trauma, mediastinal tumors
CT of the chest	Single calcifications in the lungs up to 3 mm; calcifications in the coronary arteries	Mediastinitis, mediastinal tumors, thoracic aneurysm, bronchitis, pneumonia, pleuritis, asthma, pulmonary carcinoma, active TB
Neurological evaluation		
Physical examination	No abnormalities	Obvious focal neurological deficits
Neurological consultation	No abnormalities	MS, Parkinson’s disease, clinical epilepsy, brain injury sequelae
EEG	No abnormalities	Epilepsy, encephalitis, encephalopathy
CT of the head	No abnormalities	Acute intracranial hemorrhage, large tumor, hydrocephalus, skull fracture, brain injury
MRI of the head	Elongated distal segment of the left vertebral artery with a tortuous course, compressing the ventral-left medulla oblongata and displacing it to the right; stage 1 leukoaraiosis; moderate cortical and subcortical atrophy	Thrombosis, aneurysm, small tumors, abscess, meningitis, encephalitis
Nerve conduction study	Axonal damage to the left phrenic nerve; conduction velocity within normal range	Phrenic nerve external compression
ENT and neck evaluation		
Otolaryngology consultation	No abnormalities	Herpes zoster, rhinitis, otitis, pharyngitis, foreign body, tympanic irritation, neck cyst, goiter
Psychiatric evaluation		
Psychiatry consultation	No abnormalities	Hysterical neurosis, conversion disorder, schizophrenia, acute stress reactions, malingering, personality disorders, anorexia nervosa, enuresis

After referral to our Home Nutrition Unit, immediate intervention was warranted due to severe malnutrition. Oral nutritional supplements and dietary fortification failed to improve intake because of ongoing gastrointestinal symptoms. Enteral tube feeding was therefore recommended; however, uncontrolled diaphragmatic contractions posed a high risk of aspiration and vomiting with gastric feeds. A review of the literature suggested that esophageal manometry during acute hiccup episodes often demonstrates suppressed peristalsis and transient lower esophageal sphincter relaxation^1^, compromising gastric feeding safety (1). Consequently, we proceeded with placement of a percutaneous endoscopic gastrostomy with jejunal extension (PEG-PEJ). Given the severity of malnutrition and the risk of refeeding syndrome, jejunal feeding was initiated cautiously using an oligomeric (semi-elemental) normocaloric formula via continuous pump infusion, starting at 50% of estimated energy requirements. The rate was gradually increased over 5 days to achieve 100% of calculated needs. Serum electrolytes (potassium, phosphate, magnesium) and clinical status were monitored daily during the initiation phase. Once stable, the patient transitioned to a polymeric high-protein formula (Isosource Protein 2.0), administered as 1 liter daily via continuous pump infusion at the speed of 100 mL/h, providing 2000 kcal and 100 g of protein. PPI therapy was continued throughout for GERD management. This regimen resulted in a marked nutritional response: over 6 months, the patient’s body weight increased by 15 kg, and muscle strength improved sufficiently to permit daily physical activity.

At 1 year post-placement, during a prolonged hiccup episode accompanied by nausea and vomiting, the jejunal part dislodged and retrogradely traversed the gastrointestinal tract—passing from the jejunum into the esophagus—and its distal tip protruded into the oral cavity. The patient, mistaking it for a foreign object, manually extracted the jejunal extension, fracturing its distal segment. Remarkably, he remained asymptomatic. Endoscopic evaluation confirmed the integrity of the gastric component. Following a discussion with the patient, the decision was made to continue with gastric feeding alone, without jejunal extension. An oligomeric formula was briefly used during the transition period, followed by continuation of the polymeric high-protein formula using the same continuous pump infusion method. The patient experienced occasional heartburn, which was effectively managed with PPIs. Importantly, hiccup-induced vomiting did not interfere with enteral nutrition, allowing for stable weight and continued improvement in muscle function.

## Discussion

Most hiccups are acute and self-limiting, resolving either spontaneously or with simple interventions within 48 h. Hiccups persisting beyond 48 h are classified as persistent, while those exceeding 1 month are termed intractable (3). While the overall incidence and prevalence of hiccups remain unclear, a cross-sectional study conducted over a 14-year period in the United States identified 66,124 emergency room patients who were subsequently admitted with hiccups documented as a discharge diagnosis. This translated to an average of 4,723 hospitalizations per year (SD ± 937) and an incidence rate of 632 cases per million hospital discharges. Among these patients, 895 were primarily admitted for hiccups as their main diagnosis with a rate of 8.4 per million hospital discharges (8). While this study from the Nationwide Inpatient Sample (NIS) indicated that the number of hospital admissions for hiccups as a primary diagnosis was relatively low, it was noteworthy that hiccups were documented as a secondary diagnosis in thousands of hospital admissions each year, and hiccups may not always have been recorded. Although hiccups were rarely diagnosed in hospitalized patients, this data underscored the importance of conducting a thorough investigation into this symptom (8). In the present case, two likely etiologies converged: gastroesophageal reflux disease (GERD), which manifests atypically as hiccups in ~4.5% of cases (9), and a history of carbonated alcohol intake, another recognized trigger (2, 10).

Treatment of hiccups should prioritize addressing the underlying cause whenever possible. If no specific pathology was identified or if a definitive treatment was not available, various pharmacological and non-pharmacological therapies had been proposed (3). Physical maneuvers and agents targeting dopaminergic or GABAergic pathways have been widely used, yet a recent systematic review highlighted the paucity of high-quality trials to guide practice (1). Acupuncture was proposed as a treatment for persistent and intractable hiccups, supported by several studies. However, these investigations, including four randomized controlled trials (n = 305), were limited by a lack of blinding or placebo controls, exhibited a high risk of bias, and failed to report potential side effects. This raised concerns about the true efficacy of acupuncture compared to placebo (11). The literature indicated that hypnosis (12) and upper endoscopy (13) had been explored as management strategies for persistent hiccups. Invasive procedures had been used for intractable hiccups unresponsive to medication (1). Given that vagal maneuvers could sometimes terminate hiccups (14), vagus nerve stimulation had emerged as a therapeutic option for intractable cases (15). This typically involved implantation of a vagus nerve stimulator (VNS) (16). Vagus nerve stimulation has produced complete relief in approximately 71% of seven published cases (15, 17). Phrenic nerve disruption may have been considered in patients with persistent hiccups that cause considerable discomfort or lead to significant morbidity. Temporary phrenic nerve block with long-acting anesthetic, such as bupivacaine is advised before considering permanent phrenic nerve disruption (18). An alternative approach involved the stellate ganglion’s (SG) role in the “reflex arc” of hiccups, where neural inhibition of the SG could interrupt the reflex (19). The injected drug likely spread to the vagus nerve, potentially blocking the phrenic nerves, anterior scalene muscle, and recurrent laryngeal nerves, thereby halting hiccups in these patients. Following the procedure, both the frequency and intensity of hiccups decreased, eventually leading to complete resolution in small series (20). Combining phrenic nerve block with stellate ganglion block may offer synergistic benefit (21). Additionally, a single-center randomized trial suggests that tailored breathing and water-drinking exercises may rival pharmacotherapy in efficacy and safety (22).

Although hiccups have been associated with oral intake difficulty and modest weight loss (2, 5, 23–25), no prior reports describe severe malnutrition directly attributable to intractable hiccups. Malnutrition is frequently underdiagnosed and undertreated, with profound adverse effects on physical function, mental health, and overall clinical outcomes (26).

This case report highlights that patients with a clinical diagnosis of hiccups may suffer from secondary severe malnutrition requiring nutritional support, including enteral nutrition. To our knowledge, there is currently no literature available regarding the potential routes for enteral nutrition in such cases, specifically whether feeding can be administered via gastric or intestinal access. The patient’s case demonstrated that gastric feeding was effective; however, it should be noted that feeding methods may depend on the underlying cause of the hiccup.

## Conclusion

Intractable hiccups therefore warrant not only etiological investigation and multimodal symptom management but also vigilant nutritional assessment. Tailoring enteral access to individual patient factors is essential: jejunal feeding may bypass vomiting risk in the acute phase, while standard gastric feeding can suffice once symptom control permits. Recognizing and addressing secondary complications such as malnutrition is critical to improving both short- and long-term outcomes in this challenging clinical scenario.

## Data Availability

The original contributions presented in the study are included in the article/supplementary material, further inquiries can be directed to the corresponding author/s.
